# Statins intake and risk of liver cancer: A dose–response meta analysis of prospective cohort studies

**DOI:** 10.1097/MD.0000000000007435

**Published:** 2017-07-07

**Authors:** Changhong Yi, Zhenggui Song, Maolin Wan, Ya Chen, Xiang Cheng

**Affiliations:** a Department of Interventional, Jingzhou Central Hospital; b The Second Clinical Medical College Yangtze University, Jingzhou; c Department of Hepatobiliary surgery, Union Hospital Affiliated to Tongji Medical College of Huazhong University of Science and Technology, Wuhan; d Department of Interventional, Zigui County Hospital of traditional Chinese Medicine, Yichang, Hubei Province, People's Republic of China.

**Keywords:** liver cancer, meta-analysis, prospective cohort studies, statins

## Abstract

Previous studies have indicated that statins intake was associated with liver cancer risk, but presented controversial results.

Studies in PubMed and EMBASE were searched update to February 2017 to identify and quantify the potential dose–response association between statins intake and liver cancer.

Six eligible studies involving a total of 11,8961 participants with 9530 incident cases were included in this meta-analysis. Statistically significant association was observed between increasing statins intake and liver cancer risk reduction (OR = 0.46, 95%CI: 0.24–0.68, *P* <.001). Furthermore, the summary relative risk of liver cancer for an increase of 50 cumulative defined daily dose per year was 0.86 (95%CI: 0.81–0.90, *P* <.001). Evidence of a nonlinear dose–response relationship between statins intake and liver cancer risk was found (*P* for nonlinearity <.01). Subgroups analysis indicated that statins intake was associated with a significantly risk of liver cancer risk reduction in Asia (OR = 0.44, 95%CI: 0.11–0.77, *P* <.001) and Caucasian (OR = 0.49, 95%CI: 0.36–0.61, *P* <.001). Subgroup meta-analyses in study design, study quality, number of participants, and number of cases showed consistency with the primary findings.

Additional statins intake is associated with liver cancer risk reduction.

## Abstract 2

Supplemental Digital Content is available in the text

## 1 Introduction

Liver cancer is the fifth most common cancer worldwide in men and the sixth most common cancer worldwide in women, and costs on patients, caregivers, and society that remains the most common malignancy.^[1]^ The etiology of liver cancer involves both genetic and environmental factors. According to the American Cancer Association statistics, liver cancer mortality gradually increased, the relative survival rate of liver cancer being 18%.^[2]^ Based on cancer registry data available in China, the age-standardized 5-year relative survival for liver cancer is 10.1% in 2015.^[3]^ These data reveal the poor prognosis of liver cancer, and thus to prevent the occurrence of liver cancer is essential. Previous studies investigating have showed that statins have a chemopreventive potential in the liver cancer.^[4]^

Statins are inhibitors of 3-hydroxy-3-methyl glutaryl coenzyme reductase A, which is a key enzyme in the rate-limiting step in cholesterol synthesis.^[5]^ Statins are widely prescribed in the primary and secondary prevention of heart attack, stroke, and cardiovascular disease.^[6]^ Recently, statin use has been reported to have a promising anticancer effect,^[7]^ and statin monotherapy could potentially reduce any organ and colorectal cancer-related mortality.^[8,9]^ Additionally, studies showing statin use has been found to be associated with decreased risks in hepatocellular carcinoma,^[10]^ pancreatic cancer,^[11]^ prostate cancer,^[12]^ gastric cancer,^[13]^ colorectal cancer,^[14]^ and breast cancer.^[15]^

Several meta-analyses of randomized controlled trials have examined the relationship between statin use and risk of liver cancer and have found that statin use is significantly reduce liver cancer risk.^[16–18]^ However, there is lack of study to quantitatively assess statin use in relation to liver cancer. Thus, we conducted a dose–response meta-analysis to clarify and quantitatively assess statin use and risk of liver cancer.

## 2 Methods

Our meta-analysis was conducted according to the Meta-analysis Of Observational Studies in Epidemiology (MOOSE) checklist.^[19]^ There are no ethical issues involved in our study for our data were based on published studies.

### 2.1 Search strategy

We included eligible studies to investigate the relationship between statins intake and liver cancer. To develop a flexible, nonlinear, r meta-regression model, we required that an eligible study should have been categorized into 3 or more levels. If multiple publications were available for a study, we included the longest follow-up study.

PubMed and EMBASE were searched for studies that were published update to February 2017, with keywords including “liver cancer” OR “hepatocellular” OR “hepatic” OR “intrahepatic” AND “statin.” We refer to the relevant original essays and commentary articles to determine further relevant research. Eligible study was also included through the reference lists of relevant review articles. The search strategy is shown in detail in the supplementary list S1.

### 2.2 Study selection

Two independent researchers (CY and ZS) investigated the information regarding the correlation between statin use and liver cancer: outcome was liver cancer; the relative risks (RR) at least 3 quantitative categories. Moreover, we precluded nonhuman studies, reviews, meta-analyses, editorials, and published letters. To ensure the correct identification of qualified research, the 2 researchers read the reports independently, and the disagreements were resolved through consensus by all of the researchers.

### 2.3 Data extraction

Each eligible article's information was extracted by 2 independent researchers (MW and YC). We extracted the following information: first author; publication year; mean value of age; country; study name; sex; cases and participants; the categories of statin use; and RR or odds ratio (OR). We collected the risk estimates with multivariable-adjusted.^[20]^ Quality assessment was performed according to the Newcastle-Ottawa scale for nonrandomized studies.^[21]^

### 2.4 Statistical analysis

We pooled RR estimates as the common measure of association statin use and liver cancer risk; the hazard ratio was considered equivalent to the RR.^[22]^ Any results stratified by different subgroups of statin use and liver cancer risk in any single article were treated as 2 separate reports.

Due to different cut-off points for categories in the included studies, we performed a RR with 95% confidence intervals (CI) by an increase of 50 cumulative defined daily dose per year using the method recommended by Greenland, Longnecker and Orsini and colleagues.^[23]^ The dose of statin intake used the median stain intake. If the median stain intake category was not available, the midpoint of the upper and lower boundaries was considered as the dose of each category. In addition, using restricted cubic splines (RCS) to evaluate the nonlinear association between statin intake and liver cancer risk, with 3 knots at the 10th, 50th, and 90th percentiles of the distribution. A flexible meta-regression based on RCS function was used to fit the potential nonlinear trend, and generalized least-square method was used to estimate the parameters.^[21]^ This procedure treats statin use (continuous data) as an independent variable and logRR of diseases as a dependent variable, with both tails of the curve restricted to linear. A *P* value is calculated for linear or nonlinear by testing the null hypothesis that the coefficient of the second spline is equal to zero.^[23]^

STATA software 12.0 (STATA Corp, College Station, TX) was used to evaluate the relationships between statin use and liver cancer risk. *Q* test and *I*^2^ statistic were used to assess heterogeneity among studies. The random-effect model was chosen if *P*_*Q*_ <.10 or *I*^2^>50%, otherwise, the fixed-effect mode was applied. Begg and Egger tests were done to assess the publication bias of each study. *P* <.05 was considered significant for all tests.

## 3 Results literature search results

**Figure 1:**
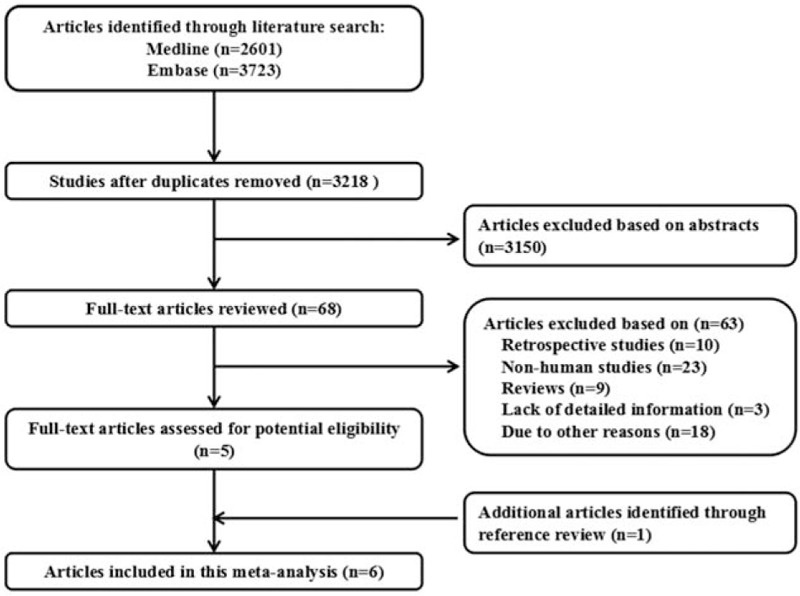
Flow diagram of the study selection process.

Figure 1 shows the results of literature research and selection. We identified 2601 articles from PubMed and 3723 articles from EMBASE. After exclusion of duplicates and studies that did not fulfill the inclusion criteria, 6 studies were chosen,^[24–29]^ and the data were extracted, and a total of 6 reports datasets were included in the final meta-analysis. These studies were published update to February 2017.

### 3.1 Study characteristics

The characteristics of the included studies are shown in the Tables 1 and 2. Among the selected studies, 6 eligible studies involving 4 cohort studies and 2 case–control studies, 2 studies are from Caucasia and 4 from Asia, a total of 11,8961 participants with 9530 incident cases were included in this meta-analysis.

**Table 1:**
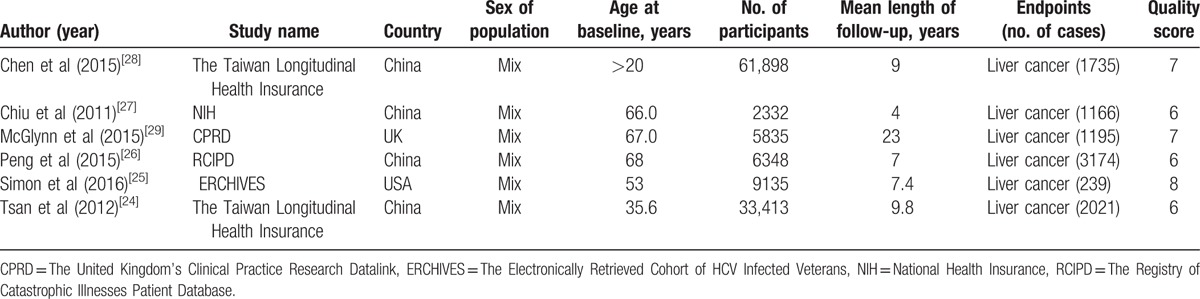
Characteristics of participants in included studies of statins intake in relation to risk of Liver cancer.

**Table 2:**
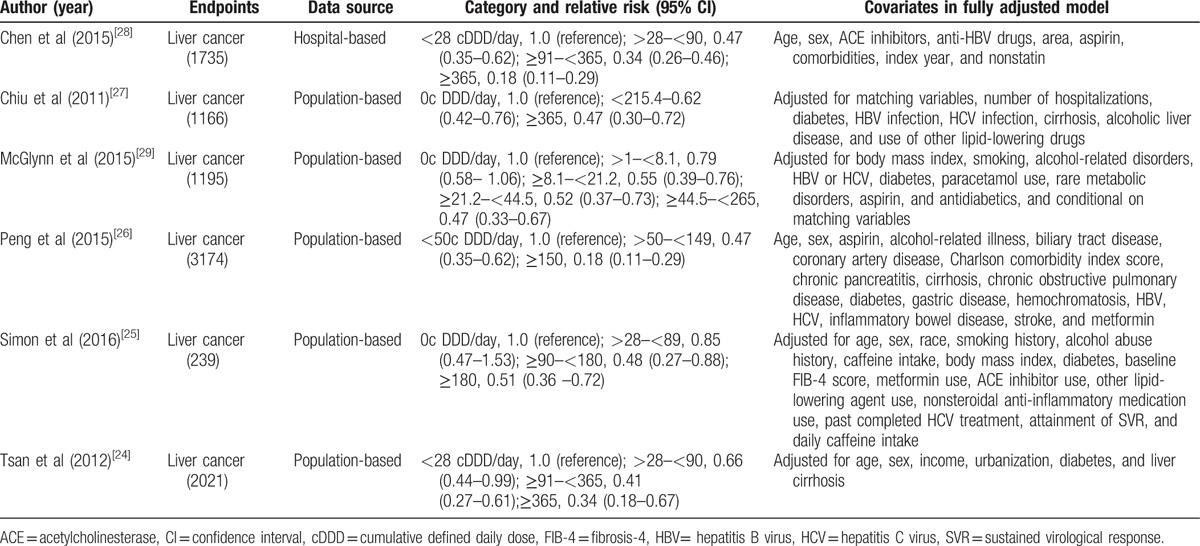
Outcomes and covariates of included studies of statins intake in relation to risk of Liver cancer.

### 3.2 Overall meta-analysis

The results of statin use and the risk of liver cancer are shown in Table 3. The pooled results suggest that statin use is significantly associated with liver cancer risk, which was suggested both by the highest and lowest categories (RR = 0.46; 95% CI: 0.24–0.68; *P* <.001) (Table 3). We found evidence of between-study heterogeneity (*I*^2^ = 91.8%, *P* <.001) but we observed no evidence of publication bias (Egger asymmetry test, *P* = .063) (Table S1).

**Table 3:**
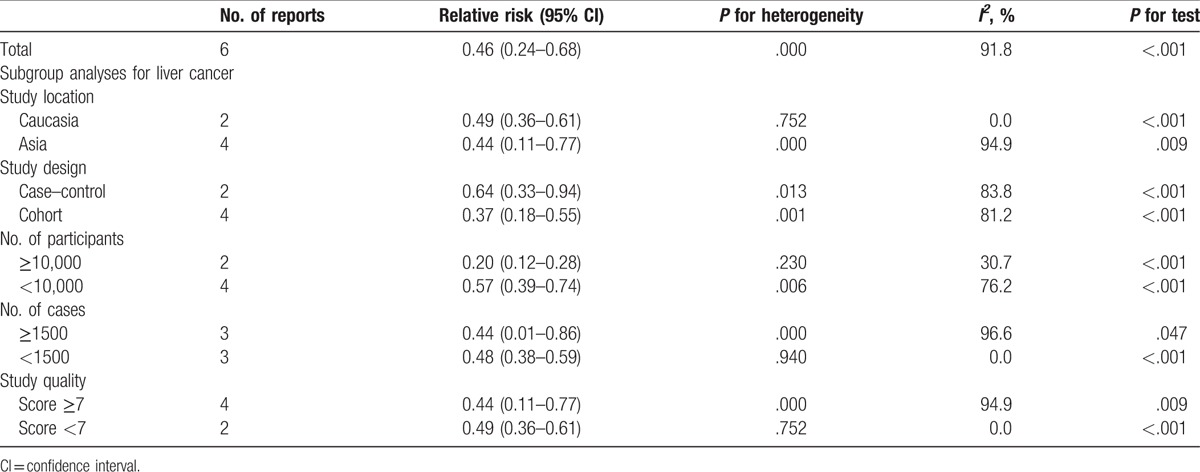
Stratified analyses of relative risk of liver cancer.

### 3.3 Dose–response meta-analyses between statins intake and liver cancer

**Figure 2:**
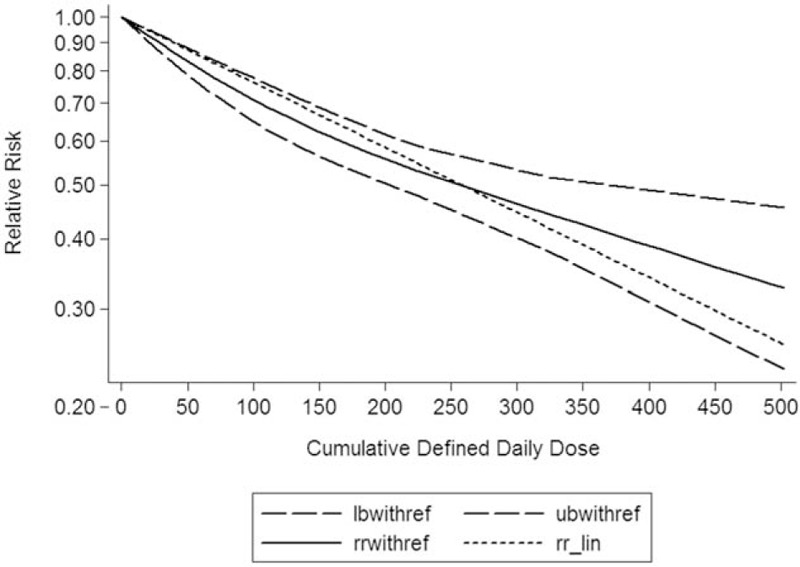
Dose–response relationship between statin intake and risk of liver cancer.

Using RCS function, the test for a nonlinear dose–response relationship was significant (likelihood ratio test, *P* <.001), suggesting curvature in the relationship, with an increase of 50 cumulative defined daily dose per year was associated with a 14% decrement in the risk of liver cancer. The summary RR of liver cancer for an increase of 50 cumulative defined daily dose per year was 0.86 (95%CI: 0.81–0.90, *P* <.001) (Fig. 2).

### 3.4 Subgroup analyses

Subgroup analysis was performed to check the stability of the primary outcome (Table 3). Subgroup analyses based on the study location found a similar risk reduction of liver cancer in Asia (OR = 0.44, 95%CI: 0.11–0.77, *P* <.001) and Caucasian (OR = 0.49, 95%CI: 0.36–0.61, *P* <.001) (Table 3). The relationship between statin use and liver cancer risk was similar in subgroup analyses, which were defined by study design, number of cases or participants, and study quality. An increment of 50 cumulative defined daily dose per year significantly decreased the liver cancer risk in any of the categories.

### 3.5 Publication bias

Each study in this meta-analysis was performed to evaluate the publication bias by both Begg funnel plot and Egger test. *P* >.05 was considered no publication bias. The results show that no obvious evidence of publication bias was found in the associations between statin use and liver cancer risk (supplementary Table S1). A funnel plot for publication bias assessment is illustrated in supplementary Figure S1.

## 4 Discussion

Statins are the most commonly used prescription drugs for the treatment of dyslipidemia. Recently, there has been an interest in a possible protective effect of statins on cancer risk,^[30]^ and statin use has been reported to have a promising anticancer effect. Statins may also have cytostatic effects that extend the survival of cancer patients.^[31]^

Statins are inhibitors of 3-hydroxy-3-methyl glutaryl coenzyme reductase A (HMG-CoA), which can combine with HMG-CoA reductase activity sites to reverse HMG-CoA reductase activity, thus inhibiting hydroxyvaleric acid synthesis, thus inhibiting several downstream products of the mevalonate pathway.^[7]^ The main substrate for statins is the protein of Ras and Rho family, plus some GTP-binding proteins such as Rab, Rac, and Ral. The main function of Rho family protein is to coordinate the movement of cells and regulate gene transcription.^[32]^ Statins inhibit the proliferation and differentiation of tumor cells by inhibiting the isoprene of Ras and Rho protein, which cannot be activated. Studies have shown that bone morphogenetic protein (BMP) pathway also has certain relationship with the incidence of tumor; statins can activate the BMP and *BMP* gene to induce cell apoptosis.^[33]^ Furthermore, statin inhibits the proteasome pathway activation, limits cell cycle-dependent kinase inhibitor p21, and p27 protein decomposition, so it plays a role of a growth inhibitor of these molecules.^[34]^

To our knowledge, several meta-analyses of observational studies and randomized controlled trials have examined the association between statin use and risk of liver cancer.^[16–18]^ However, no study has been done to quantitatively assess statin use in relation to liver cancer. This is the first study to quantify the potential dose–response association between statin use and risk of liver cancer in a large cohort of both men and women. The primary finding in our meta-analysis is that statin use is significantly associated with liver cancer risk; an increase of 50 cumulative defined daily dose per year was associated with a 14% decrement in the risk of liver cancer. Subgroup analysis also proved the stability of the primary outcome. Previously it was hypothesized that the highest category of statins may have a greater chemoprotective effect in liver cancer, but in our hypothesis an increase of 50 cumulative defined daily dose per year was associated with a 14% decrement in the risk of liver cancer.

Although, we performed this meta-analysis very carefully, however, some limitations must be considered in the current meta-analysis. First, different sex of population should be included in this meta-analysis to explore the impact of different sex of population on statin use and liver cancer. Second, we only select literature that was written in English, which may have resulted in a language or cultural bias, other language should be chosen in further study. Third, there might be insufficient statistical power to check the association.

In conclusion, our meta-analysis suggests that statin use was independently associated with deleterious liver cancer risk reduction. However, large sample size, different ethnic, and different sex population of population are warranted to validate this association.

## Supplementary Material

Supplemental Digital Content
