# Supplementary material for: Statins intake and risk of liver cancer: A dose–response meta analysis of prospective cohort studies
Source: Medicine (Baltimore). 2017 Jul 7;96(27):e7435. doi: 10.1097/MD.0000000000007435 (PMC5502182; doi:10.1097/MD.0000000000007435)
Supplement: Supplemental Digital Content [file medi-96-e7435-s001.docx]

**Supplementary figure 1. Funnel of statins use and liver cancer risk.**

**
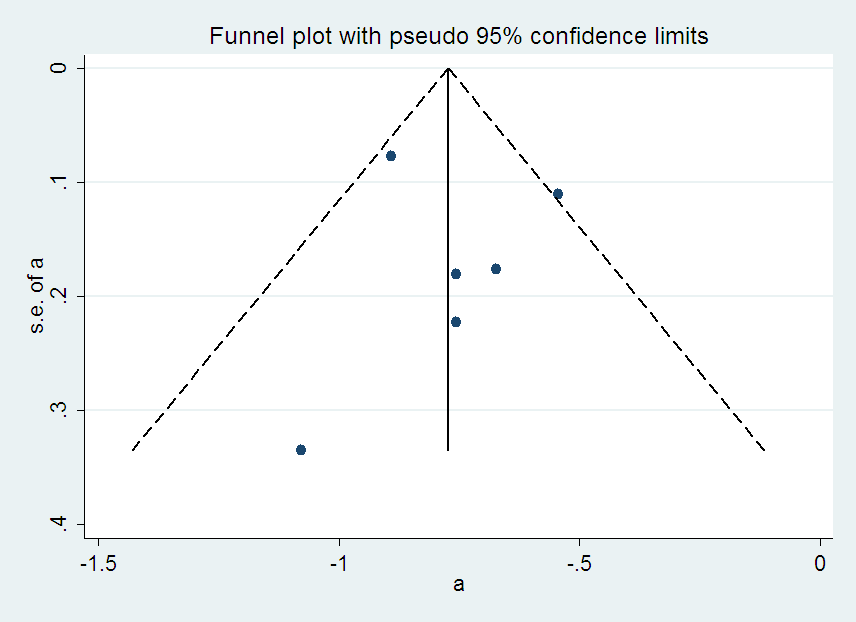
**

**Supplementary table 1. Publication bias analysis of the meta-analysis**


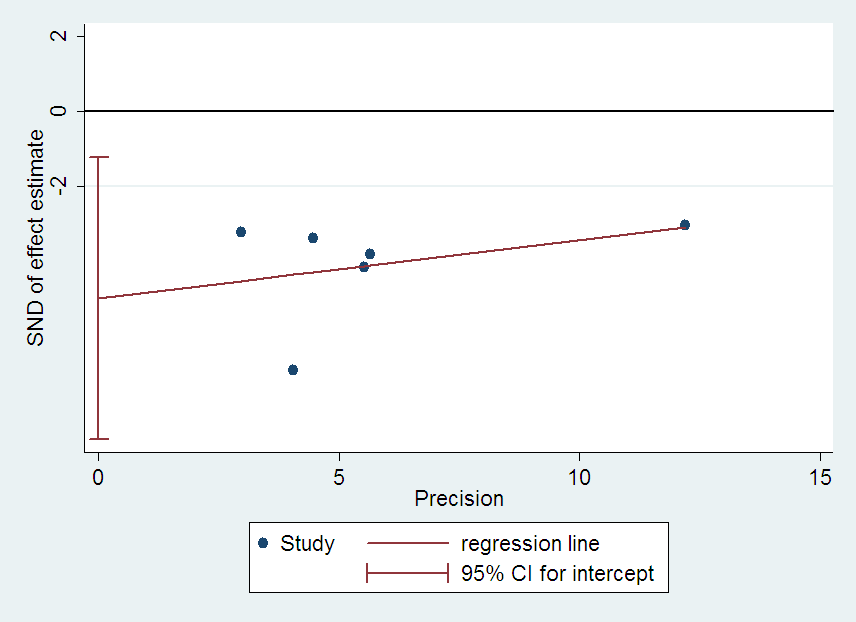


|  | **Test** | **t** | **95% CI** | **P** |
| --- | --- | --- | --- | --- |
| **Statins intake** | Begg’s test |  |  | 0.071 |
|  | Egger’s test | -3.68 | -8.76,-1.23 | 0.063 |

**Supplementary List 1 Search Strategy**

**PubMed**

#1 (((cancer*[Title/Abstract]) OR neoplasm*[Title/Abstract]) OR carcinoma*[Title/Abstract]) OR malignanc*[Title/Abstract]

#2 ((liver[Title/Abstract]) OR hepatic[Title/Abstract]) OR hepatocellular[Title/Abstract]

#3 #1 AND #2

#4 ((hepatocarcinoma[Title/Abstract]) OR "liver cell carcinoma*"[Title/Abstract]) OR hepatoma[Title/Abstract]

#5 #3 OR #4

#6 ((((((((((statin*[Title/Abstract]) OR "HMG-CoA reductase inhibitor*"[Title/Abstract]) OR "Hydroxymethylglutaryl-CoA reductase

inhibitor*"[Title/Abstract]) OR atorvastatin[Title/Abstract]) OR cerivastatin[Title/Abstract]) OR fluvastatin[Title/Abstract]) OR

lovastatin[Title/Abstract]) OR pravastatin[Title/Abstract]) OR rosuvastatin[Title/Abstract]) OR simvastatin[Title/Abstract]) OR pitavastatin[Title/Abstract]

#7 #5 AND #6

**EMBASE**

#1 statin* AND ([article]/lim OR [article in press]/lim OR [conference abstract]/lim OR [conference paper]/lim) AND ([chinese]/lim OR

[english]/lim) AND [embase]/lim

#2 'hmg coa' AND reductase AND inhibitor* AND ([article]/lim OR [article in press]/lim OR [conference abstract]/lim OR [conference

paper]/lim) AND ([chinese]/lim OR [english]/lim) AND [embase]/lim

#3 'hydroxymethylglutaryl coa' AND reductase AND inhibitor* AND ([article]/lim OR [article in press]/lim OR [conference abstract]/lim

OR [conference paper]/lim) AND ([chinese]/lim OR [english]/lim) AND [embase]/lim

#4 atorvastatin AND ([article]/lim OR [article in press]/lim OR [conference abstract]/lim OR [conference paper]/lim) AND ([chinese]/lim

OR [english]/lim) AND [embase]/lim

#5 cerivastatin AND ([article]/lim OR [article in press]/lim OR [conference abstract]/lim OR [conference paper]/lim) AND ([chinese]/lim

OR [english]/lim) AND [embase]/lim

#6 fluvastatin AND ([article]/lim OR [article in press]/lim OR [conference abstract]/lim OR [conference paper]/lim) AND ([chinese]/lim

OR [english]/lim) AND [embase]/lim

#7 lovastatin AND ([article]/lim OR [article in press]/lim OR [conference abstract]/lim OR [conference paper]/lim) AND ([chinese]/lim OR

[english]/lim) AND [embase]/lim

#8 pravastatin AND ([article]/lim OR [article in press]/lim OR [conference abstract]/lim OR [conference paper]/lim) AND ([chinese]/lim

OR [english]/lim) AND [embase]/lim

#9 rosuvastatin AND ([article]/lim OR [article in press]/lim OR [conference abstract]/lim OR [conference paper]/lim) AND ([chinese]/lim

OR [english]/lim) AND [embase]/lim

#10 simvastatin AND ([article]/lim OR [article in press]/lim OR [conference abstract]/lim OR [conference paper]/lim) AND ([chinese]/lim

OR [english]/lim) AND [embase]/lim

#11 pitavastatin AND ([article]/lim OR [article in press]/lim OR [conference abstract]/lim OR [conference paper]/lim) AND ([chinese]/lim

OR [english]/lim) AND [embase]/lim

#12 #1 OR #2 OR #3 OR #4 OR #5 OR #6 OR #7 OR #8 OR #9 OR #10 OR #11

#13 cancer* AND ([article]/lim OR [article in press]/lim OR [conference abstract]/lim OR [conference paper]/lim) AND ([chinese]/lim OR

[english]/lim) AND [embase]/lim

#14 neoplasm* AND ([article]/lim OR [article in press]/lim OR [conference abstract]/lim OR [conference paper]/lim) AND ([chinese]/lim

OR [english]/lim) AND [embase]/lim

#15 carcinoma* AND ([article]/lim OR [article in press]/lim OR [conference abstract]/lim OR [conference paper]/lim) AND ([chinese]/lim

OR [english]/lim) AND [embase]/lim

#16 malignanc* AND ([article]/lim OR [article in press]/lim OR [conference abstract]/lim OR [conference paper]/lim) AND ([chinese]/lim

OR [english]/lim) AND [embase]/lim

#17 #13 OR #14 OR #15 OR #16

#18 liver AND ([article]/lim OR [article in press]/lim OR [conference abstract]/lim OR [conference paper]/lim) AND ([chinese]/lim OR

[english]/lim) AND [embase]/lim

#19 hepatic AND ([article]/lim OR [article in press]/lim OR [conference abstract]/lim OR [conference paper]/lim) AND ([chinese]/lim OR

[english]/lim) AND [embase]/lim

#20 hepatocellular AND ([article]/lim OR [article in press]/lim OR [conference abstract]/lim OR [conference paper]/lim) AND

([chinese]/lim OR [english]/lim) AND [embase]/lim

#21 #18 OR #19 OR #20

#22 #17 AND #21

#23 hepatocarcinoma AND ([article]/lim OR [article in press]/lim OR [conference abstract]/lim OR [conference paper]/lim) AND

([chinese]/lim OR [english]/lim) AND [embase]/lim

#24 liver AND cell AND carcinoma* AND ([article]/lim OR [article in press]/lim OR [conference abstract]/lim OR [conference paper]/lim)

AND ([chinese]/lim OR [english]/lim) AND [embase]/lim

#25 hepatoma AND ([article]/lim OR [article in press]/lim OR [conference abstract]/lim OR [conference paper]/lim) AND ([chinese]/lim

OR [english]/lim) AND [embase]/lim

#26 #23 OR #24 OR #25

#27 #22 OR #26

#28 #12 AND #27
